# Identification of microRNA hsa-miR-30c-5p as an inhibitory factor in the progression of hepatocellular carcinoma and investigation of its regulatory network via comprehensive analysis

**DOI:** 10.1080/21655979.2021.1979439

**Published:** 2021-09-21

**Authors:** Shangshang Hu, Jinyan Zhang, Xiaoyu Fang, Guoqing Guo, Jing Dai, Zhiyong Sheng, Dongdong Li, Jiasheng Chen, Li Zhang, Chuanmiao Liu, Yu Gao

**Affiliations:** a School of Laboratory Medicine, Research Center of Clinical Laboratory Science, School of Laboratory Medicine, Bengbu Medical College, Bengbu, China; b School of Life Science, Bengbu Medical College, Bengbu, China; c Anhui Province Key Laboratory of Translational Cancer Research, Bengbu Medical College, Bengbu, China; d Department of Infectious Diseases, First Affiliated Hospital of Bengbu Medical College, Bengbu Medical College, Bengbu, China; e National Clinical Research Center for Infectious Diseases, First Affiliated Hospital of Bengbu Medical College, Bengbu Medical College, Bengbu, China

**Keywords:** Hsa-miR-30c-5p, hepatocellular carcinoma, miRNA, lncRNAs, ceRNA regulatory network, inhibitory factor

## Abstract

Hepatocellular carcinoma (HCC) is a primary liver cancer with high morbidity and mortality. An increasing number of abnormal gene expressions were identified to be associated with the progression of HCC. Previous studies showed that the hsa-miR-30 c-5p (miR-30 c), one of the miR-30 family members, might play a role in suppressing tumor progression in a variety of tumors. The present study aims to examine miR-30 c effects in the development of HCC. The role of miR-30 c in HCC was comprehensively investigated by using bioinformatics and experiments *in vitro*. The multiple databases were combined to predict and screen the target genes and upstream lncRNAs of miR-30 c, and then constructed a competitive endogenous RNA (ceRNA) regulatory network with miR-30 c as the central miRNA. The miR-30 c-related ceRNA regulatory network was also initially validated *in vitro*. The results showed that miR-30 c over-expression could inhibit proliferation, migration, invasion, induce apoptosis, and increase G0/G1 phase ratio of HCC cells. Three miR-30 c upstream lncRNAs and 12 miR-30 c target genes were expressed in HCC cells with increased expression and poor prognosis, and a miR-30 C-related ceRNA regulatory network was constructed. This study verified miR-30 c as an inhibitory factor in the progression of HCC and performed analyses on the miR-30 c regulatory network, which might provide potential target information for HCC prognoses and therapies. However, further experiments *in vivo* and studies including clinical trials will be conducted to validate our results.

## 1. Introduction

Primary liver cancer is one of the most common malignancies, and about 80% of patients are hepatocellular carcinoma (HCC), the fourth highest mortality rate of cancer worldwide [1]. In recent years, treatments for HCC have been greatly developed, but these treatments do not have enough effect on advanced HCC [2]. It is well known that that abnormal gene expression might be associated with the development of HCC. To improve the diagnosis and treatment of HCC, it is crucial to identify and validate prognostic biomarkers for HCC.

MicroRNA (miRNA) is a class of endogenous, single-stranded, non-coding RNA of 19–25 nt length, in which its prominent role is to bind to the 3ʹ untranslated region of its target mRNA, thereby inhibiting gene expression [3]. The interactions of mRNA, miRNA, and lncRNA (long-stranded non-coding RNA) play multiple roles in the development of HCC [4]. Interestingly, two recent studies have provided tremendous convenience for detecting aberrantly expressed miRNAs and mRNAs in the early stage of tumors [5,6]. A growing number of studies have shown that competitive endogenous RNA (ceRNA) regulatory networks might play important roles during HCC process [7]. The miR-30 c, one of the miR-30 family members, is closely associated with the physiological activities of a variety of tumors. The previous studies reported that miR-30 c might play a role in inhibiting tumor progressions, such as prostate cancer [8], gastric cancer [9], breast cancer [10], thyroid cancer [11], and glioblastoma [12].

In this study, we hypothesized that miR-30 c acts as a tumor suppressor promote HCC development via a miR-30 c-centered ceRNA regulatory network. The present study aimed to explore the role of miR-30 c in the progression of HCC by using bioinformatics tools and *in vitro* experiments. At first, the multiple databases were combined to predict and screen the target genes and upstream lncRNAs of miR-30 c. After that, a miR-30 c-centered ceRNA regulatory network related to HCC prognosis was constructed based on the list of mRNAs and lncRNAs. Finally, the miR-30 c-related ceRNA regulatory network was initially validated *in vitro*.

## 2. Materials and methods

### 2.1. Data source

Public datasets were analyzed in this study. The data can be found in the Gene Expression Omnibus database (GEO database, https://www.ncbi.nlm.nih.gov/geo), and The Cancer Genome Atlas (TCGA database, https://portal.gdc.cancer.gov). The patients involved in the database have obtained ethical approval. Users can download relevant data for free for research and publish relevant articles. This study is based on open-source data, so there are no ethical issues and other conflicts of interest.

### 2.2. Bioinformatics analysis

Expression profile data for HCC were obtained from the TCGA database, including mRNA/lncRNA (tumor:n = 374, normal:n = 50) and miRNA (tumor:n = 375, normal:n = 50), and 50 pairs of HCC samples. The datasets of GSE147889 (tumor:n = 97, normal:n = 97) and GSE10694 (tumor:n = 78, normal:n = 88) were obtained from the GEO database. The Kaplan-Meier survival curves of HCC patients with miR-30 c expression were plotted using the miRNA module in Kaplan-Meier Plotter (http://www.kmplot.com) [13]. The miRNet online tool was used to predict the upstream lncRNA of miR-30 c [14]. Target genes to miR-30 c were predicted by using DIANA [15], mirwalk [16], TargetScan [17], and miRDB [18] online tools and databases. And then, combined with TCGA liver cancer expression profiles and two GEO datasets (GSE45267 and GSE84402) expression profiles, the upregulated miR-30 c target genes in HCC were identified (logFC ≥ 0 and P < 0.05). Based on the expression profiles and survival data, the upstream lncRNA of miR-30 c and the screened miR-30 c downstream target genes were subjected to one-way cox analysis using the R software survival package to identify the lncRNAs and miR-30 c target genes associated with poor prognosis in HCC patients.

### 2.3. Cell culture and qRT-PCR

Five cell lines, including four HCC cells (SMMC-7721, SNU449, MHCC97-H, and HUH-7) and immortalized human hepatocyte WRL68 cells, were cultured in the complete medium consisted of 1% double antibodies (streptomycin and penicillin), 10.0% fetal bovine serum, and 89% DMEM basal medium, at 37°C in a 95% air-5% CO_2_ mixture. Total RNA was extracted using MiPure® cell miRNA KIT (Novozymes Biotechnology, China). In this kit, total RNA or miRNA could be extracted and separated by using different silica gel columns. Here, total RNAs, including mRNAs, lncRNAs and miRNAs, were extracted by using MiPure® RNAspin Column (Novozymes Biotechnology, China). U6 was used as the internal reference gene for amplification of miRNA, and GAPDH was used as the internal reference gene for amplification of mRNA. The LightCycler®96 real-time PCR system was used for qRT-qPCR detection of the amplified products. The primer sequences were listed in Supplementary Table 1.

**Table 1.** The summary of the role of miRNA-30 c in human cancer

Type of cancer	Samples	Role of miR-30 c [Reference]
Hepatocellular carcinoma	Cell lines	Inhibition of HCC development [22]
Hepatocellular carcinoma	the whole blood	Diagnostic markers [30]
Hepatocellular carcinoma	Tissues and Cell lines	Prognostic markers [20]
Prostate cancer	Cell lines	Inhibition of prostate cancer development [23]
Prostate cancer	Tissues and Cell lines	Inhibition of prostate cancer development [24]
Prostate cancer	Tissues and Cell lines	Inhibition of prostate cancer development [8]
Gastric cancer	Tissues and Cell lines	Inhibition of gastric cancer development [9]
Glioblastoma	Tissues and Cell lines	Inhibition of glioblastoma development [12]
Breast cancer	Tissues and Cell lines	Inhibition of breast cancer development [27]
Breast Cancer	Cell lines	To enhance breast cancer chemosensitivity [26]
Pancreatic Ductal Adenocarcinoma	Tissues and Cell lines	Inhibition of pancreatic ductal adenocarcinoma development [29]
Pancreatic cancer	Tissues and Cell lines	Inhibition of pancreatic cancer development [28]
Clear cell renal cell carcinoma	Urine and Cell lines	Inhibition of clear cell renal cell carcinoma development [31]

### 2.4. Transient transfection

According to the sequence of miR-30 c (UGUA AACAU CCUACACUCUCAGC), miR-30 c mimic (UUACAUUUGUAGGAUGUGAGAGU) and mimic-NC (ACGUGACACGUUCGGAGAATT) were synthesized by GeneCopeia^TM^ (Guangzhou, China). When the cell density reached 70%, the cells were transiently transfected according to the transfection reagent EndoFectin^TM^-MAX instructions (GeneCopoeia^TM^, Guangzhou, China). After 48 h, transfected cells were collected for experimental analyses.

### 2.5. Cell cycle assay

The cells were digested with trypsin to make cell suspension, centrifuged to remove supernatant, washed twice with ice precooled PBS, and then fixed with pre-chilled 70% ethanol for 12–24 h. After that, ethanol was removed by centrifugation. And then, the cells were washed twice with ice precooled PBS again. Finally, the cells were resuspended with propidium iodide (PI) and incubated in the dark at 37°C for 30 min. Flow cytometry analysis was performed in a FACSCalibur flow cytometer (Backmancoulter, USA).

### 2.6. Cell apoptosis assay

The cells were digested with trypsin into single cell suspension. The cells were washed once with ice precooled PBS, and were resuspended in PBS. After that, the supernatant was removed by centrifugation, and cells were resuspended by adding 195uL Annexin V-FITC binding solution, 5 μL of Annexin V-FITC, and 10 μL of propidium iodide (PI), and then incubated for 20 minutes at room temperature in the dark. After that, the samples were detected using a FACSCalibur flow cytometer (Backmancoulter, USA) immediately.

### 2.7. Cell counting kit-8 (CCK8) analysis

The cells were seeded in 96-well plates with 200uL of medium containing 2 × 10^3^ cells per well, with five replicates per group. The samples were assayed at 24 h, 48 h, 72 h, and 96 h, respectively. Twenty uL of Cell counting kit-8 (CCK8) reagent was added to each well, followed by an additional 3 hours of incubation at 37 ^o^C. Subsequently, the absorbance was measured at 450 nm using a Multiskan FC microplate reader (Thermo Fisher Scientific).

### 2.8. Colony formation assay

The cells (1000 cells/well) were seeded in 6-well plates and cultured for two weeks. After the medium was aspirated, the cells were fixed with 4% paraformaldehyde for 20 min, followed by staining with crystal violet for 15 min, and finally rinsing gently twice with PBS. A spot with more than 50 cells was counted as one clone.

### 2.9. Cell scratch healing assay

The cells were cultured in 6-well plates. When the cells formed 80% of monolayer cells, a 10uL pipette tip was used to draw a blank area on the confluent monolayer of the cells. The cells were gently rinsed twice using PBS, followed by the addition of a complete culture medium. Photographs were taken at 0 h, 24 h, and 48 h after scratching. The migration rate was calculated as the following formula: ‘24 h migration rate = (0 h scratch area – 24 h scratch area)/0 h scratch area’, and ‘48 h migration rate = (0 h scratch area – 48 h scratch area)/0 h scratch area’.

### 2.10. Transwell migration and invasion assay

The migration and invasion abilities of cells were measured using Transwell chambers (model: 3422; Costar, USA). Cells were prepared in a serum-free medium and starved overnight. A certain number of cells (200 uL of serum-free medium containing 3 × 10^4^ cells in migration assay; 200 uL of serum-free medium containing 4 × 10^4^ cells in invasion assay) were counted the next day and inoculated in the Transwell. In the lower chamber, 600 uL of medium containing 10% serum was added. The invasion assay was performed using Matrigel (BD Biosciences) coating. After culturing for 24 h at 37°C, the samples were fixed with 4% paraformaldehyde and were stained with 1% crystal violet. The number of migrated and invaded cells were counted in 5 random fields of view.

### 2.11. Statistical analysis

All the above analyses were carried out by using R software version 4.0.3. Independent samples t-test and paired t-test were used for TCGA data and GEO data. The data from experiments *in vitro* were obtained from three independent experiments (mean ± standard deviation), and the independent samples t-test was used for statistical analyses. Statically significance was defined as P < 0.05 (*), P < 0.01 (**), P < 0.001 (***), and P < 0.0001 (****).

## 3. RESULTS

In the current study, the role of miR-30 c in HCC progression was investigated, and the impact of a miR-30 c-centered ceRNA regulatory network was predicted during the development of HCC. The expression profile of miR-30 c was significantly lower in HCC tissues by comprehensive analysis using multiple databases. Based on TCGA data, HCC patients with lower miR-30 c expression levels had a poor prognosis. The over-expression of miR-30 c could block G0/G1 cycle, promote apoptosis of HCC cells in vitro, and inhibit proliferation, migration, and invasion of HCC cells in vitro. The target genes and upstream lncRNAs of miR-30 c were predicted and identified based on multiple databases, and a miR-30 c-centered ceRNA regulatory network related to HCC prognosis was constructed. Further, the miR-30 c-centered ceRNA regulatory network was preliminarily validated by using in vitro experiments. Taken together, the results suggested that miR-30 c acts as a tumor suppressor promote HCC development via a miR-30 c-centered ceRNA regulatory network.

### 3.1. Downregulation of miR-30 c is observed in HCC tissues and cell lines

**Figure 1.**
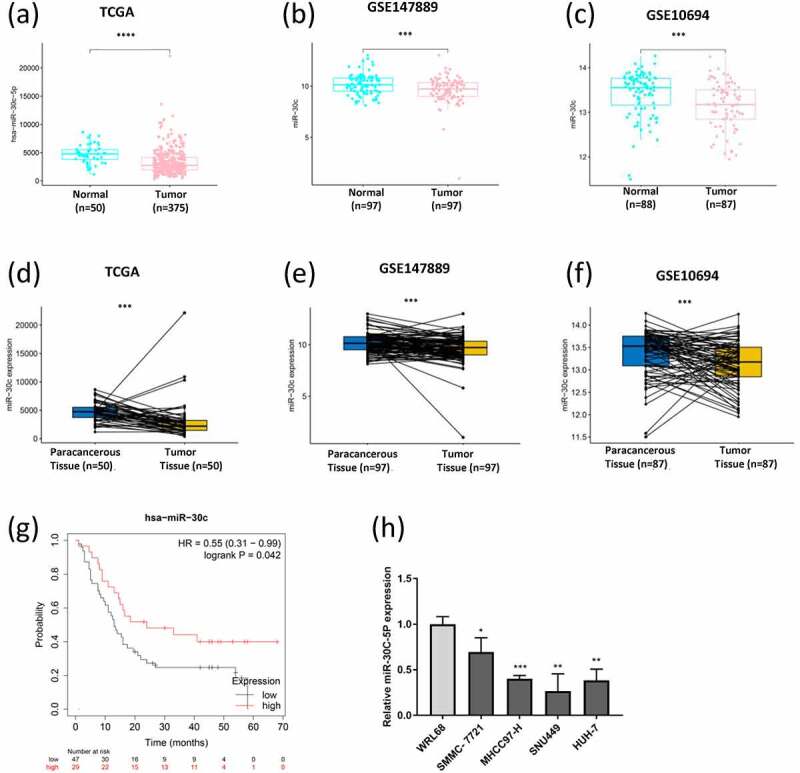
Role of miR-30 c in hepatocellular carcinoma (HCC). Differential expression of miR-30 c in HCC based on TCGA data (a) and (d). Differential expression of miR-30 c in HCC based on the GSE147889 dataset (b) and (e), and the GSE10694 dataset (c) and (f). (g) Effect of miR-30 c expression on the overall survival of HCC patients (based on Kaplan-Meier Plotter). (h) Differential expression of miR-30 c in four HCC cells (SMMC-7721, SNU449, MHCC97-H, and HUH-7) and immortalized human hepatocyte WRL68 cells

The significant downregulations of miR-30 c expression were observed in HCC tissues according to the data from TCGA data (Figure 1(a)), GSE147889 dataset (Figure 1(b)), and GSE10694 dataset (Figure 1(c)) by using bioinformatics tools. Moreover, the miR-30 c expression was dramatically lower in HCC tissues compared with their paracarcinoma tissues (Figure 1(d), 1(e), and 1(f)). Kaplan-Meier Plotter results showed that the low expression of miR-30 c was correlated with poor overall survival time of HCC patients (Figure 1(g)). Moreover, the miR-30 c expression was significantly lower in four HCC cell lines (SMMC-7721, SNU449, MHCC97-H, and HUH-7) compared with that in human liver cell line WRL68 (Figure 1(h)).

### 3.2. miR-30 c blocks G0/G1 cycle and promotes apoptosis of HCC cells in vitro

**Figure 2.**
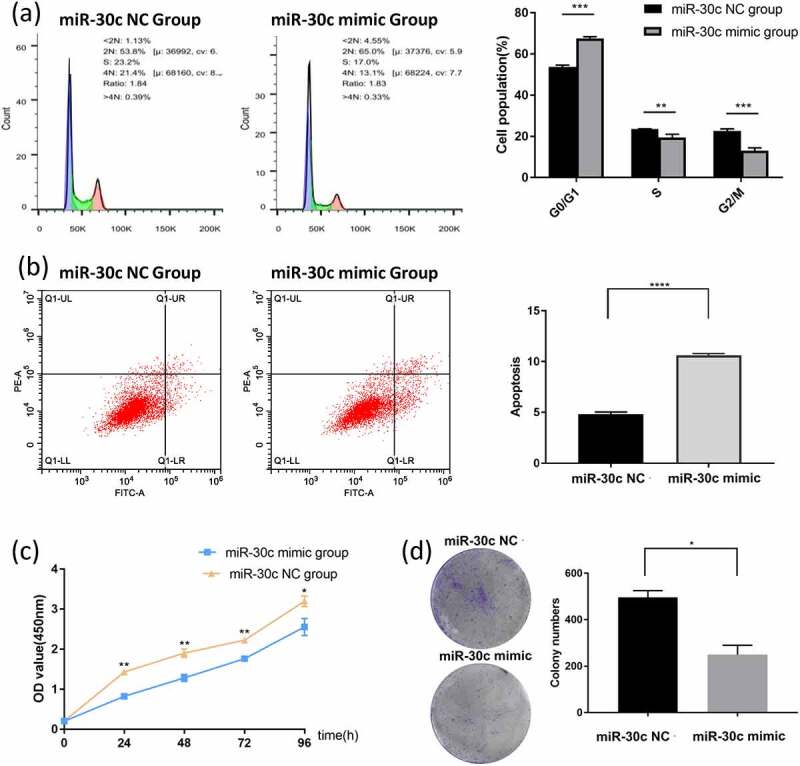
Overexpression of miR-30 c induces SNU449 cell cycle arrest and apoptosis, and inhibits SNU449 cell proliferation. (a) Effect of overexpression of miR-30 c on SNU449 cell cycle. (b) Effect of overexpression of miR-30 c on apoptosis in SNU449 cells. (c) CCK8 assay detects SNU449 cell proliferation. (d) Colony formation assay detects SNU449 cell proliferation

SNU449 cells, which showed the lowest expression level of miR-30 c, were transfected with miR-30 c mimic to overexpress miR-30 c. The transfection efficiency of miR-30 c mimic in SNU449 cells was measured by qRT-PCR (Supplementary Figure 1). Compared with the miR-30 c NC group, miR-30 c overexpression blocked SNU449 cells in the G0/G1 phase, while the number of cells in the S and G2/M phases was significantly higher in the miR-30 c NC group than in the miR-30 c mimic group (Figure 2(a)). By calculating the percentage of early apoptotic and late apoptotic cells (UR and LR regions), the percentage of apoptotic cells in the miR-30 c mimic group was significantly higher than that in the miR-30 c NC group (Figure 2(b)). Therefore, miR-30 c overexpression could block the SNU449 G0/G1 cell cycle and promote apoptosis in SNU449 cells.

### 3.3. miR-30 c inhibits proliferation of HCC cells in vitro

The effect of miR-30 c on the proliferation of HCC cells was explored by miR-30 c mimic transfection of SNU449 cells. The proliferation ability of the miR-30 c mimic group was significantly smaller than that of the miR-30 c NC group at each period of 24 h, 48 h, 72 h, and 96 h (Figure 2(c)). The colony formation assay further showed that miR-30 c overexpression could inhibit the proliferation ability of SNU449 cells (Figure 2(d)). The results indicated that miR-30 c could inhibit the proliferation of SNU449 cells.

### 3.4. *miR-30 c inhibits migration and invasion of HCC* cells *in vitro*

**Figure 3.**
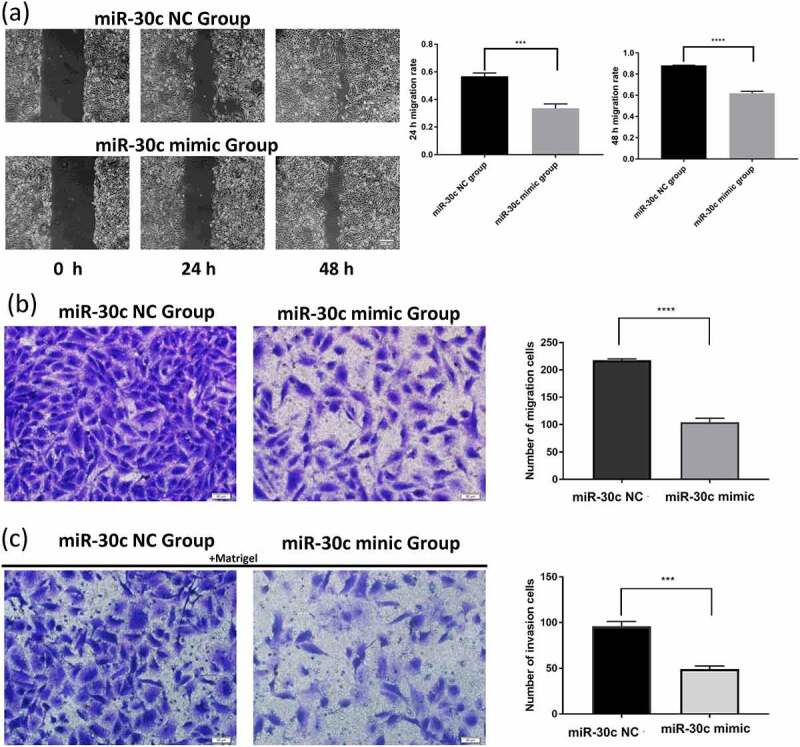
Overexpression of miR-30 c inhibits SNU449 cell migration and invasion. (a) Scratch assay and (b) Transwell assay to detect SNU449 cell migration ability. (c) Transwell assay (containing matrix gel) to detect SNU449 cell invasion ability

To investigate whether miR-30 c affects the migration and invasion of HCC cells, the effect of miR-30 c on migration and invasion of SNU449 cells was detected by scratch assay and Transwell assay. The scratch assay results showed that miR-30 c overexpression inhibited the migration ability of SNU449 cells at 24 h and 48 h (Figure 3(a)). To further validate the results of the scratch assay, Transwell assays were performed. In the Transwell migration assay, overexpression of miR-30 c significantly reduced the number of migrating SNU449 cells (Figure 3(b)). Results of the Transwell invasion assay also showed (Figure 3(c)) that overexpression of miR-30 c reduced the number of SNU449 cells passing through the stromal gel. Taken together, miR-30 c could affect the migration and invasion of SNU449 cells.

### 3.5. Construction of a miR-30 c-centered ceRNA regulatory network associated with HCC prognosis

**Figure 4.**
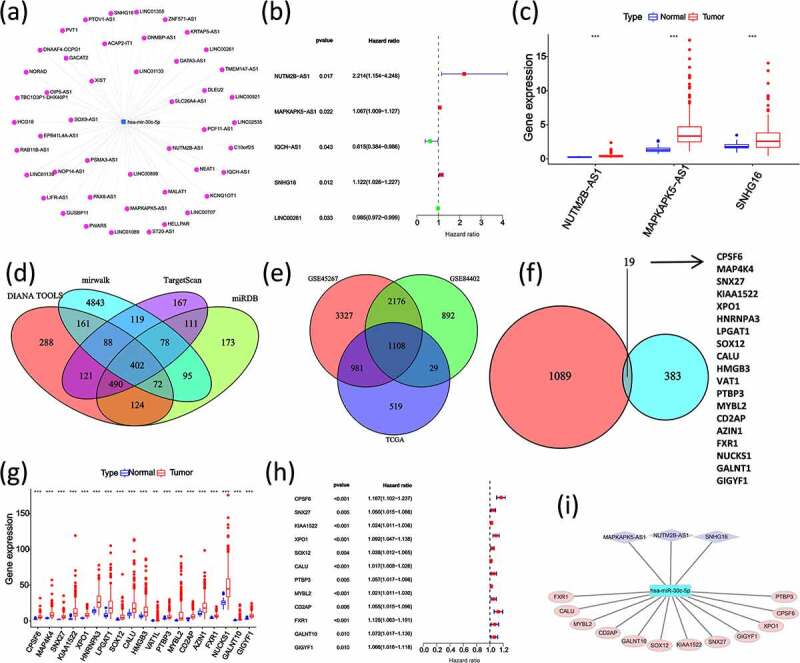
Construction of miR-30 c-centered ceRNA regulatory network with the identifications of miR-30 c upstream and downstream lncRNA and mRNA with high expression and poor prognosis. (a) Prediction of miR-30 c upstream lncRNAs by miRNet database. (b) Screening of poor prognosis lncRNAs in miR-30 c upstream. (c) Differential expression of poor prognosis lncRNAs measured in HCC tissues. (d) DIANA, mirwalk, TargetScan, and miRDB predicted target genes downstream of miR-30 c. (e) TCGA data, GSE45267 dataset, and GSE84402 dataset expression profiles were screened for genes upregulated in HCC (logFC≥0 and P < 0.05). (f) Screening of miR-30 c target genes upregulated in HCC. (g) Differential expression of miR-30 c target genes in HCC tissues. (h) Screening of miR-30 c target genes associated with poor prognosis in HCC. (i) Construction of miR-30 c-centered ceRNA regulatory network

The above results showed that miR-30 c was lowly expressed in HCC tissues and HCC patients with lower miR-30 c expression levels had a poor prognosis. According to that, miR-30 c was used as the center to identify lncRNAs and mRNAs with high expression levels and poor prognoses, respectively. A total of 46 upstream lncRNAs of miR-30 c were predicted by using miRNet database (Figure 4(a)). Three lncRNAs, including NUTM2B antisense RNA 1 (NUTM2B-AS1), MAPKAPK5 antisense RNA 1 (MAPKAPK5-AS1) and small nucleolar RNA host gene 16 (SNHG16), were subsequently screened out because of the association with poor prognosis of HCC on one-way Cox analysis (Figure 4(b)) and with the higher expression levels in HCC tissues (Figure 4(c)). Four online databases (DIANA, mirwalk, TargetScan, and miRDB) were applied to predict the target genes downstream of miR-30 c. A total of 402 miR-30 c target genes were identified (Figure 4(d)). Based on the expression profiles of TCGA HCC data, GSE45267 dataset, and GSE84402 dataset, 1108 genes were identified with high expression levels in HCC (Figure 4(e)). Then, 19 miR-30 c target genes, also upregulated in HCC, were identified (Figure 4(f)). Subsequently, the one-way Cox analysis, which was performed on these 19 upregulated miR-30 c target genes, showed that 12 genes had poor prognoses in HCC, including cleavage polyadenylation specific factor 6 (CPSF6), sorting nexin 27 (SNX27), uncharacterized protein KIAA1522, exportin 1 (XPO1), SRY-box transcription factor 12 (SOX12), calumenin (CALU), polypyrimidine tract binding protein 3 (PTBP3), MYB proto-oncogene like 2 (MYBL2), CD2 associated protein (CD2AP), FMR1 autosomal homolog 1 (FXR1), polypeptide N-acetylgalactosaminyltransferase 10 (GALNT10), and GRB10 interacting GYF protein 1 (GIGYF1) (Figure 4(g) and 4(h)). Finally, a ceRNA regulatory network centered on miR-30 c and associated with HCC prognosis was constructed (Figure 4(i)).

### 3.6. Preliminary validation of the miR-30 c-centered ceRNA regulatory network

**Figure 5.**
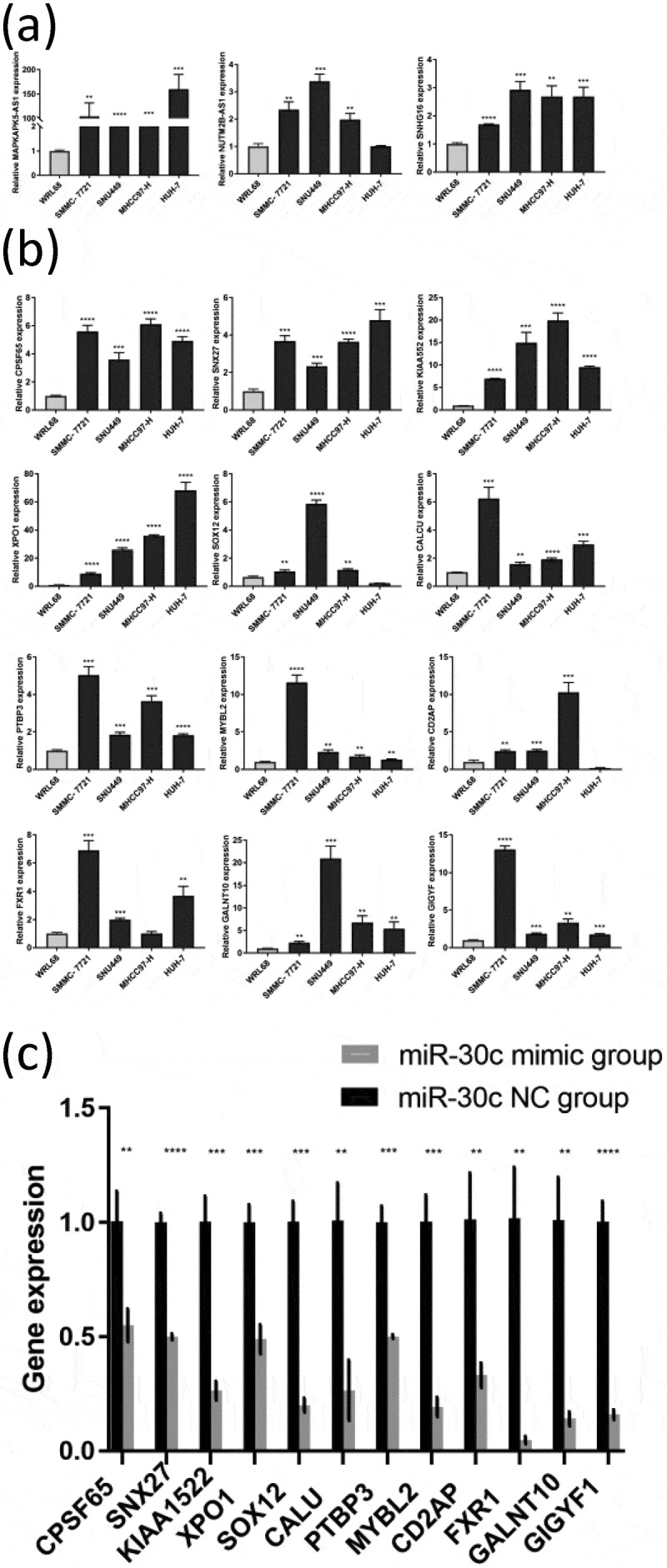
Preliminary validation of miR-30 c-centered ceRNA regulatory network. (a) The lncRNAs upstream of miR-30 c (NUTM2B-AS1, MAPKAPK5-AS1, and SNHG16) were highly expressed in HCC cell lines. (b) Differential expression of 12 miR-30 c target genes (CPSF6, SNX27, KIAA1522, XPO1, SOX12, CALU, PTBP3, MYBL2, CD2AP, FXR1, GALNT10, and GIGYF1) in HCC cell lines. (c) After transfection with miR-30 c mimic, the expression levels of 12 miR-30 c target genes were down-regulated

The result of the miR-30 c-centered ceRNA regulatory network was preliminarily validated by using qRT-PCR. The results showed that three miR-30 c upstream lncRNAs (NUTM2B-AS1, MAPKAPK5-AS1, and SNHG16) were highly expressed in four HCC cell lines (except for NUTM2B-AS1 in the HUH-7 cell line) (Figure 5(a)). As shown in Figure 5(b), SOX12 and CD2AP were lowly expressed in the HUH-7 cell line but highly expressed in the other three HCC cell lines (SMMC-7721, SNU449, and MHCC97-H). Moreover, the other 10 miR-30 c downstream target genes (CPSF6, SNX27, KIAA1522, XPO1, CALU, PTBP3 MYBL2, FXR1, GALNT10, and GIGYF1) were highly expressed in all four HCC cell lines. Subsequently, miR-30 c mimic transfection of SNU449 cells was used to explore whether miR-30 c overexpression could affect the expression of those 12 miR-30 c downstream target genes. As shown in Figure 5(c), the expression levels of all 12 miR-30 c target genes were down-regulated after transfection of SNU449 cells with miR-30 c mimic. Taken together, three miR-30 c upstream lncRNAs and 12 miR-30 c target genes were highly expressed in HCC cells, and overexpression of miR-30 c could affect the expression of 12 miR-30 c target genes.

## 4. Discussion

HCC is a primary liver cancer with high morbidity and mortality. It is crucial to elucidate the mechanism of HCC development and identify valuable biomarkers and therapeutic targets. Aberrant expression of lncRNA, miRNA, and mRNA and their interactions play important roles in HCC [19]. To the best of our knowledge, no miR-30 c-centered ceRNA regulatory network has been established in HCC. Although miR-30 c has not been studied so much in HCC, the previous study showed that HCV (hepatitis C virus) core protein could attenuate miR-30 c expression in HCC, and further studies showed that downregulation of miR-30 c could activate epithelial-mesenchymal transition (EMT) [20]. Another study showed that serum miR-30 c expression was significantly dysregulated in cirrhosis and HCC patients, so that miR-30 c might be used as a novel noninvasive biomarker for HCV-positive cirrhosis and HCC [21].

In this study, the role of miR-30 c in HCC was analyzed using bioinformatics and experiments *in vitro*. The results showed that miR-30 c expression was down-regulated in HCC cell lines, and overexpression of miR-30 c could inhibit proliferation, migration, and invasion of HCC cells. The findings in this study were very similar to the previous report, which indicated the effect of miR-30 c on the migration and invasion of SMMC-7721 and HepG2 cells [22]. Comparing results of this work with already published works, the expression levels of miRNA-30 c were all decreased in the different type of cancers (Table 1). MiRNA-30 c could suppress prostate cancer survival by targeting KRAS Proto-Oncogene [23], E2F Transcription Factor 7 (E2F7) [8], or alternative splicing factor/splicing factor 2 (ASF/SF2) [24]. In breast cancer progression, miRNA-30 c also play a role by inhibiting KRAS signaling [25], histone deacetylase 9 (HDAC9) [26], or coactosin-like protein 1 (COTL1) [27]. In the development and progression of pancreatic cancer, miR-30 c had vital functions by targeting twinfilin 1 (TWF1) [28] or DNA topoisomerase II alpha gene (TOP2A) [29]. Because the expression of miR-30 c was significantly down-regulated in the plasma of HCC samples, it might be used as a noninvasive diagnostic marker [30]. The urinary exosome miR-30 c was also suggested as a biomarker of clear cell renal cell carcinoma [31].

According to multiple databases, three lncRNAs (NUTM2B-AS1, MAPKAPK5-AS1, and SNHG16) and 12 mRNAs (CPSF6, SNX27, KIAA1522, XPO1, SOX12, CALU, PTBP3, MYBL2, CD2AP, FXR1, GALNT10, and GIGYF1) were identified to associate with miR-30 c, which were upregulated-expressed in HCC and had poor prognosis in HCC patients. Among them, NUTM2B-AS1 has not been studied in HCC before, and it is the first time to identify that its expression is upregulated in HCC cell lines. There are several reports on the roles of SNHG16 gene in HCC, and the results showed that SNHG16 could promote proliferation, migration, and invasion of HCC cells [32,33], and it also might act as a ceRNA targeting miRNA to promote HCC development, such as miR-186 [34] and miR-4500 [35]. In a recent study, MAPKAPK5-AS1 was found to be act as a ceRNA to upregulate PLAG1 like zinc finger 2 (PLAGL2) expression in miR-154-5p to promote HCC development [36]. Among the 12 miR-30 c target genes reported in this paper, SNX27, CALU, CD2AP, FXR1, and GIGYF1 have not been studied in HCC. Here, we demonstrated their high expression levels in HCC cell lines, which were consistent in HCC samples through bioinformatics analyses. Several studies have shown that seven other miR-30 c target genes, including CPSF6 [37], KIAA1522 [38], XPO1 [39], SOX12 [40], PTBP3 [41], MYBL2 [42], and GALNT10 [43] had a pro-developmental effect on HCC.

In this study, a ceRNA regulatory network with the center of miR-30 c was constructed and preliminarily validated. The expression of 12 downstream target genes of miR-30 c was downregulated after overexpression of miR-30 c. In this miR-30 c-centered ceRNA regulatory network, each RNA might play an important prognostic role in HCC process. Based on the contents of this study and the results of other published studies, the role of miR-30 c as an oncogenic factor was validated by using SNU449 HCC cells. MiR-30 c overexpression could inhibit proliferation, migration, and invasion of HCC cells, and cause cell cycle arrest and induce apoptosis in HCC cells. In addition, we constructed a miR-30 c-centered ceRNA regulatory network related to the prognosis of HCC, and extrapolated that the miR-30 c ceRNA regulatory network might be closely associated with the development of HCC. It is hoped that the present findings could provide a theoretical basis for elucidating the mechanism of miR-30 c in HCC progression, and the results could provide data support that miR-30 c might be a potential therapeutic target for the treatment of HCC.

## 5. Conclusion

In this study, we confirmed that the expression level of miR-30 c was identified lower in HCC cells and constructed a miR-30 c-centered ceRNA regulatory network related to HCC prognosis. The overexpression of miR-30 c could inhibit HCC cells proliferation, migration, and invasion. The results demonstrated for the first time that miR-30 c might play a role during the process of the pathogenesis in HCC via the ceRNA regulatory network. The result suggested that miR-30 c might be a potential therapeutic target for the treatment of HCC. However, further experiments *in vivo* and studies including clinical trials will be conducted to validate our results.

## Supplementary Material

Supplemental Material

## Data Availability

Publicly available datasets were analyzed in this study, these can be found in GEO database (https://www.ncbi.nlm.nih.gov/geo), and The Cancer Genome Atlas (https://portal.gdc.cancer.gov). The authors confirm that the data supporting the findings of this study are available within the article and its supplementary materials.

## Highlights

(1) miR-30c overexpression could inhibit HCC cells proliferation, migration and invasion

(2) a miR-30c ceRNA regulatory network might be associated with the development of HCC

(3) miR-30c might be a potential therapeutic target for the treatment of HCC

## References

[1] Bray F, Ferlay J, Soerjomataram I, et al. Global cancer statistics 2018: GLOBOCAN estimates of incidence and mortality worldwide for 36 cancers in 185 countries. CA Cancer J Clin. 2018;68(6):394–424. 30207593 10.3322/caac.21492

[2] Xu W, Liu K, Chen M, et al. Immunotherapy for hepatocellular carcinoma: recent advances and future perspectives. Ther Adv Med Oncol. 2019;11:1758835919862692. 31384311 10.1177/1758835919862692 PMC6651675

[3] Wang L, Ge S, Zhou F. MicroRNA-487a-3p inhibits the growth and invasiveness of oral squamous cell carcinoma by targeting PPM1A. Bioengineered. 2021;12(1):937–947. 33724144 10.1080/21655979.2021.1884396 PMC8291853

[4] Liao Z, Zhang H, Su C, et al. Long noncoding RNA SNHG14 promotes hepatocellular carcinoma progression by regulating miR-876-5p/SSR2 axis. J Exp Clin Cancer Res. 2021;40(1):36. 33485374 10.1186/s13046-021-01838-5 PMC7824933

[5] Shin Low S, Pan Y, Ji D, et al. Smartphone-based portable electrochemical biosensing system for detection of circulating microRNA-21 in saliva as a proof-of-concept. Sens Actuators B Chem. 2020;308:127718.

[6] Prasad KS, Cao X, Gao N, et al. A low-cost nanomaterial-based electrochemical immunosensor on paper for high-sensitivity early detection of pancreatic cancer. Sens Actuators B Chem. 2020;305:127516. 32863588 10.1016/j.snb.2019.127516 PMC7453835

[7] Long J, Bai Y, Yang X, et al. Construction and comprehensive analysis of a ceRNA network to reveal potential prognostic biomarkers for hepatocellular carcinoma. Cancer Cell Int. 2019;19(1):90. 31007608 10.1186/s12935-019-0817-y PMC6458652

[8] Wang Y, Pei X, Xu P, et al. E2F7, regulated by miR‑30c, inhibits apoptosis and promotes cell cycle of prostate cancer cells. Oncol Rep. 2020;44(3):849–862. 32582990 10.3892/or.2020.7659 PMC7388350

[9] Han W, Mu Y, Zhang Z, et al. Expression of miR-30c and BCL-9 in gastric carcinoma tissues and their function in the development of gastric cancer. Oncol Lett. 2018;16(2):2416–2426. 30013632 10.3892/ol.2018.8934 PMC6036597

[10] Yuan RX, Bao D, Zhang Y. Linc00707 promotes cell proliferation, invasion, and migration via the miR-30c/CTHRC1 regulatory loop in breast cancer. Eur Rev Med Pharmacol Sci. 2020;24(9):4863–4872. 32432749 10.26355/eurrev_202005_21175

[11] Xue C, Cheng Y, Wu J, et al. Circular RNA CircPRMT5 accelerates proliferation and invasion of papillary thyroid cancer through regulation of miR-30c/E2F3 axis. Cancer Manag Res. 2020;12:3285–3291. 32494192 10.2147/CMAR.S249237 PMC7231777

[12] Liu S, Li X, Zhuang S. miR-30c Impedes glioblastoma cell proliferation and migration by targeting SOX9. Oncol Res. 2019;27(2):165–171. 29495977 10.3727/096504018X15193506006164 PMC7848431

[13] Menyhárt O, Nagy Á, Győrffy B. Determining consistent prognostic biomarkers of overall survival and vascular invasion in hepatocellular carcinoma. R Soc Open Sci. 2018;5(12):181006. 30662724 10.1098/rsos.181006 PMC6304123

[14] Chang L, Zhou G, Soufan O, et al. miRNet 2.0: network-based visual analytics for miRNA functional analysis and systems biology. Nucleic Acids Res. 2020;48(W1):W244–w251. 32484539 10.1093/nar/gkaa467 PMC7319552

[15] Paraskevopoulou MD, Georgakilas G, Kostoulas N, et al. DIANA-microT web server v5.0: service integration into miRNA functional analysis workflows. Nucleic Acids Res. 2013;41(WebServer issue):W169–173. 23680784 10.1093/nar/gkt393 PMC3692048

[16] Shin C, Nam JW, Farh KK, et al. Expanding the microRNA targeting code: functional sites with centered pairing. Mol Cell. 2010;38(6):789–802. 20620952 10.1016/j.molcel.2010.06.005 PMC2942757

[17] Agarwal V, Bell GW, Nam JW, et al. Predicting effective microRNA target sites in mammalian mRNAs. Elife. 2015,Aug 12;4:e05005. doi: 10.7554/eLife.05005. PMID: 26267216; PMCID: PMC4532895. PMC4532895 26267216

[18] Chen Y, Wang X. miRDB: an online database for prediction of functional microRNA targets. Nucleic Acids Res. 2020;48(D1):D127–d131. 31504780 10.1093/nar/gkz757 PMC6943051

[19] Xu G, Xu WY, Xiao Y, et al. The emerging roles of non-coding competing endogenous RNA in hepatocellular carcinoma. Cancer Cell Int. 2020;20(1):496. 33061848 10.1186/s12935-020-01581-5 PMC7552539

[20] Liu D, Wu J, Liu M, et al. Downregulation of miRNA-30c and miR-203a is associated with hepatitis C virus core protein-induced epithelial-mesenchymal transition in normal hepatocytes and hepatocellular carcinoma cells. Biochem Biophys Res Commun. 2015;464(4):1215–1221. 26210453 10.1016/j.bbrc.2015.07.107

[21] Oksuz Z, Serin MS, Kaplan E, et al. Serum microRNAs; miR-30c-5p, miR-223-3p, miR-302c-3p and miR-17-5p could be used as novel non-invasive biomarkers for HCV-positive cirrhosis and hepatocellular carcinoma. Mol Biol Rep. 2015;42(3):713–720. 25391771 10.1007/s11033-014-3819-9

[22] Wu W, Zhang X, Liao Y, et al. miR-30c negatively regulates the migration and invasion by targeting the immediate early response protein 2 in SMMC-7721 and HepG2 cells. Am J Cancer Res. 2015;5(4):1435–1446. 26101708 PMC4473321

[23] Zhang J, Wang X, Wang Y, et al. Low expression of microRNA-30c promotes prostate cancer cells invasion involved in downregulation of KRAS protein. Oncol Lett. 2017;14(1):363–368. 28693177 10.3892/ol.2017.6163 PMC5494817

[24] Huang YQ, Ling XH, Yuan RQ, et al. miR30c suppresses prostate cancer survival by targeting the ASF/SF2 splicing factor oncoprotein. Mol Med Rep. 2017;16(3):2431–2438. 28677791 10.3892/mmr.2017.6910 PMC5548014

[25] Tanic M, Yanowsky K, Rodriguez-Antona C, et al. Deregulated miRNAs in hereditary breast cancer revealed a role for miR-30c in regulating KRAS oncogene. PLoS One. 2012;7(6):e38847. 22701724 10.1371/journal.pone.0038847 PMC3372467

[26] Liang Z, Feng A, Shim H. MicroRNA-30c-regulated HDAC9 mediates chemoresistance of breast cancer. Cancer Chemother Pharmacol. 2020;85(2):413–423. 31907648 10.1007/s00280-019-04024-9

[27] Pei B, Li T, Qian Q, et al. Downregulation of microRNA-30c-5p was responsible for cell migration and tumor metastasis via COTL1-mediated microfilament arrangement in breast cancer. Gland Surg. 2020;9(3):747–758. 32775265 10.21037/gs-20-472 PMC7347814

[28] Sun LL, Cheng M, Xu XD. MicroRNA-30c inhibits pancreatic cancer cell proliferation by targeting twinfilin 1 and indicates a poor prognosis. World J Gastroenterol. 2019;25(42):6311–6321. 31754292 10.3748/wjg.v25.i42.6311 PMC6861845

[29] Tanaka T, Okada R, Hozaka Y, et al. Molecular pathogenesis of pancreatic ductal adenocarcinoma: impact of miR-30c-5p and miR-30c-2-3p regulation on oncogenic genes. Cancers (Basel). 2020;12(10):10. 10.3390/cancers12102731 PMC7598296 32977589

[30] Wong VC, Wong MI, Lam CT, et al. Hallmark microRNA signature in liquid biopsy identifies hepatocellular carcinoma and differentiates it from liver metastasis. J Cancer. 2021;12(15):4585–4594. 34149922 10.7150/jca.59933 PMC8210546

[31] Song S, Long M, Yu G, et al. Urinary exosome miR-30c-5p as a biomarker of clear cell renal cell carcinoma that inhibits progression by targeting HSPA5. J Cell Mol Med. 2019;23(10):6755–6765. 31342628 10.1111/jcmm.14553 PMC6787446

[32] Xie X, Xu X, Sun C, et al. Long intergenic noncoding RNA SNHG16 interacts with miR-195 to promote proliferation, invasion and tumorigenesis in hepatocellular carcinoma. Exp Cell Res. 2019;383(1):111501. 31306653 10.1016/j.yexcr.2019.111501

[33] Hu YL, Feng Y, Chen YY, et al. SNHG16/miR-605-3p/TRAF6/NF-κB feedback loop regulates hepatocellular carcinoma metastasis. J Cell Mol Med. 2020;24(13):7637–7651. 32436333 10.1111/jcmm.15399 PMC7339162

[34] Chen H, Li M, Huang P. LncRNA SNHG16 promotes hepatocellular carcinoma proliferation, migration and invasion by regulating mir-186 expression. J Cancer. 2019;10(15):3571–3581. 31293662 10.7150/jca.28428 PMC6603422

[35] Lin Q, Zheng H, Xu J, et al. LncRNA SNHG16 aggravates tumorigenesis and development of hepatocellular carcinoma by sponging miR-4500 and targeting STAT3. J Cell Biochem. 2019;120(7):11604-11615. 10.1002/jcb.28440 30779219

[36] Wang L, Sun L, Liu R, et al. Long non-coding RNA MAPKAPK5-AS1/PLAGL2/HIF-1α signaling loop promotes hepatocellular carcinoma progression. J Exp Clin Cancer Res. 2021;40(1):72. 33596983 10.1186/s13046-021-01868-z PMC7891009

[37] Tan S, Zhang M, Shi X, et al. CPSF6 links alternative polyadenylation to metabolism adaption in hepatocellular carcinoma progression. J Exp Clin Cancer Res. 2021;40(1):85. 33648552 10.1186/s13046-021-01884-z PMC7923339

[38] Jiang S, Zhang Y, Li Q, et al. KIAA1522 promotes the progression of hepatocellular carcinoma via the activation of the wnt/β-catenin signaling pathway. Onco Targets Ther. 2020;13:5657–5668. 32606779 10.2147/OTT.S251157 PMC7305824

[39] Chen L, Huang Y, Zhou L, et al. Prognostic roles of the transcriptional expression of exportins in hepatocellular carcinoma. Biosci Rep. 2019;39(8):8. 10.1042/BSR20190827 PMC6702357 31371628

[40] Zhang W, Liu K, Liu S, et al. MicroRNA‑744 inhibits migration and invasion of hepatocellular carcinoma cells by targeting SOX12. Oncol Rep. 2018;40(6):3585–3592. 30542724 10.3892/or.2018.6774

[41] Yang X, Qu S, Wang L, et al. PTBP3 splicing factor promotes hepatocellular carcinoma by destroying the splicing balance of NEAT1 and pre-miR-612. Oncogene. 2018;37(50):6399–6413. 30068940 10.1038/s41388-018-0416-8

[42] Guan Z, Cheng W, Huang D, et al. High MYBL2 expression and transcription regulatory activity is associated with poor overall survival in patients with hepatocellular carcinoma. Curr Res Transl Med. 2018;66(1):27–32. 29274707 10.1016/j.retram.2017.11.002

[43] Wu Q, Liu HO, Liu YD, et al. Decreased expression of hepatocyte nuclear factor 4α (Hnf4α)/microRNA-122 (miR-122) axis in hepatitis B virus-associated hepatocellular carcinoma enhances potential oncogenic GALNT10 protein activity. J Biol Chem. 2015;290(2):1170–1185. 25422324 10.1074/jbc.M114.601203 PMC4294483

