# Supplementary material for: Identification of microRNA hsa-miR-30c-5p as an inhibitory factor in the progression of hepatocellular carcinoma and investigation of its regulatory network via comprehensive analysis
Source: Bioengineered. 2021 Sep 21;12(1):7154–66. doi: 10.1080/21655979.2021.1979439 (PMC8806565; doi:10.1080/21655979.2021.1979439)
Supplement: Supplemental Material [file KBIE_A_1979439_SM3573.zip › supplementary/Supplementary Table 1.docx]

**Supplementary Table 1.** Primer sequences for qRT-PCR

| RNA name | Primer sequence 5’-3’ |
| --- | --- |
| GAPDH | Forward primer：ATTGAAAATTCAGGATGGGCTTTT |
|  | Reverse primer：GTTTCTGGGCTTCTCTTTGGACTC |
| U6 | Forward prime：CTCGCTTCGGCAGCACA |
|  | Reverse primer：AACGCTTCACGAATTTGCGT |
| miR-30c-5p | Forward prime：TGTAAACATCCTACACTCTCAGCAA |
|  | Reverse primer：GCTGTCAACGATACGCTACGTAACG |
| NUTM2B-AS1 | Forward primer：GCTGAGCCCTGATCTCTATGCTTT |
|  | Reverse primer：CTCTGCCTCCTCCTCTTGCTTC |
| MAPKAPK5-AS1 | Forward primer：TCTGAGAGGATCTGTGGAGGTCA |
|  | Reverse primer：GCCAAACGGCTACCTTGTAAAGA |
| SNHG16 | Forward primer：GCGTTCTTTGGGCTTCATCTTTA |
|  | Reverse primer：CAAGACGGCATCCTGTGTAACTG |
| CPSF6 | Forward primer：GGCGTGGACCACATAGACATT |
|  | Reverse primer：CCATGTAATCTCGGTCTTCTGGG |
| SNX27 | Forward primer：AGTGACGACTCGTTGGGACA |
|  | Reverse primer：TGTATCTGGGGACCGATATGG |
| KIAA1522 | Forward primer：CCAGGACAACGTCTTCTTTCC |
|  | Reverse primer：CAGCCACCCTTGTTCAGTTTC |
| XPO1 | Forward primer：ATCTGACCCAACTTGTGTAGAGA |
|  | Reverse primer：TGGTCCTACTTGCTCCAACAAT |
| SOX12 | Forward primer：AAGAGGCCGATGAACGCATT |
|  | Reverse primer：TAGTCCGGGTAATCCGCCAT |
| CALU | Forward primer：AATAGACGCGGATAAAGATGGGT |
|  | Reverse primer：GCCATTGGTTTTCAACATTGTCA |
| PTBP3 | Forward primer：GCTGTCAGTGCCGTCCAAT |
|  | Reverse primer：AGGGTAACAGGGTAAAAGAGGT |
| MYBL2 | Forward primer：CCGGAGCAGAGGGATAGCA |
|  | Reverse primer：CAGTGCGGTTAGGGAAGTGG |
| CD2AP | Forward primer：TGTGAAACTTCGGACAAGAACA |
|  | Reverse primer：AGTGACTGTAGGATTAAGGGCT |
| FXR1 | Forward primer：CTGCGACAGATTGGTTCTAGG |
|  | Reverse primer：TGTACCATAACCGGAGGTGTAA |
| GALNT10 | Forward primer：GAGCTGGTCGCCGAGATTG |
|  | Reverse primer：CCCTTCCCGTTTCTTGGTTC |
| GIGYF1 | Forward primer：AACCTGCTCCCGACGATGA |
|  | Reverse primer：AGGCTGAGGTATGTACGTCCC |
