# Supplementary figures and images for: Identification of microRNA hsa-miR-30c-5p as an inhibitory factor in the progression of hepatocellular carcinoma and investigation of its regulatory network via comprehensive analysis
Source: Bioengineered. 2021 Sep 21;12(1):7154–66. doi: 10.1080/21655979.2021.1979439 (PMC8806565; doi:10.1080/21655979.2021.1979439)

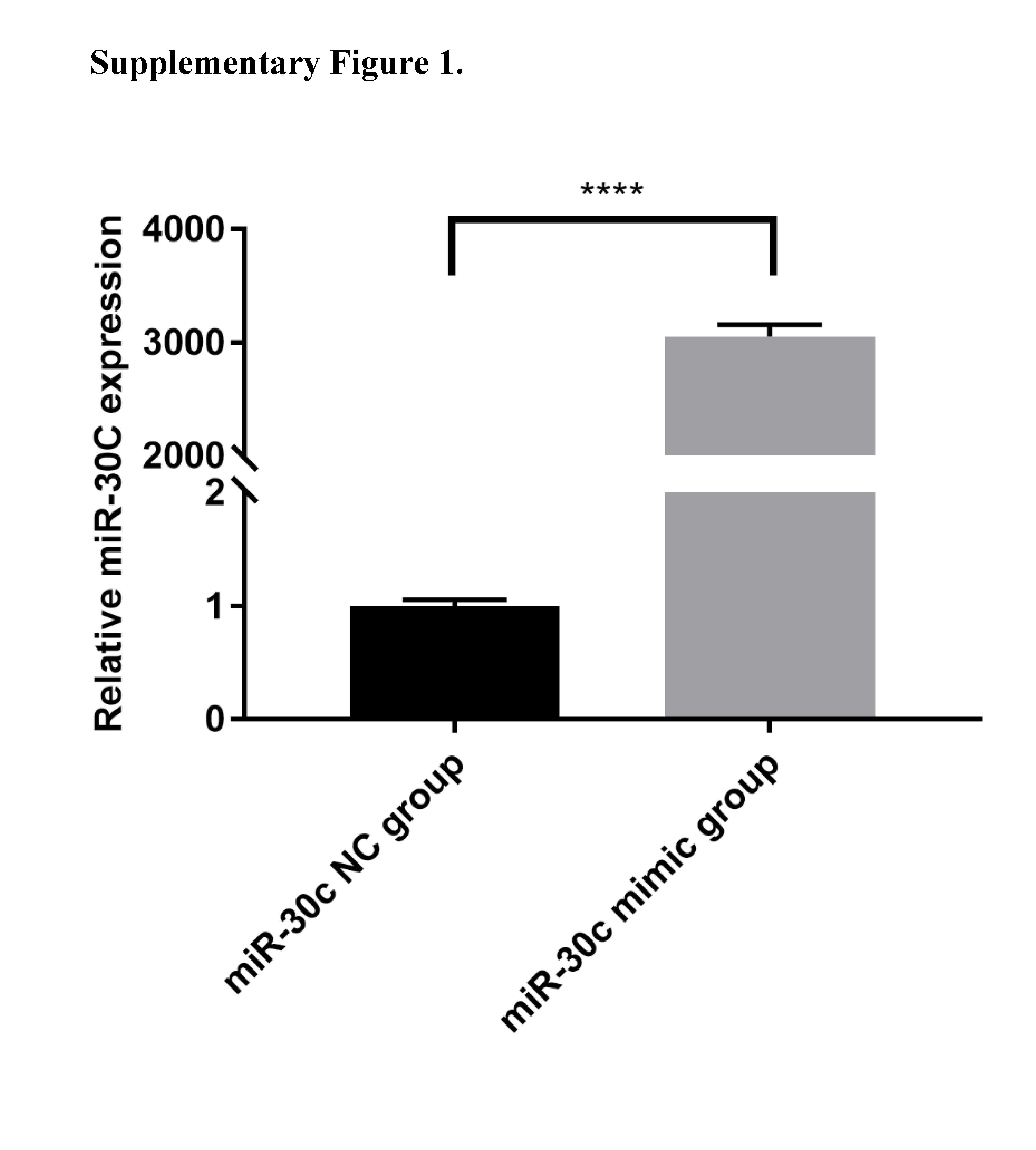

Supplement: Supplemental Material [file KBIE_A_1979439_SM3573.zip › supplementary/Supplementary Figure 1.tiff]
